# Relationship between Fear Conditionability and Aversive Memories: Evidence from a Novel Conditioned-Intrusion Paradigm

**DOI:** 10.1371/journal.pone.0079025

**Published:** 2013-11-14

**Authors:** Melanie Wegerer, Jens Blechert, Hubert Kerschbaum, Frank H. Wilhelm

**Affiliations:** 1 Division of Clinical Psychology, Psychotherapy, and Health Psychology, Department of Psychology, University of Salzburg, Salzburg, Austria; 2 Department of Cell Biology, University of Salzburg, Salzburg, Austria; Radboud University, Netherlands

## Abstract

Intrusive memories – a hallmark symptom of posttraumatic stress disorder (PTSD) – are often triggered by stimuli possessing similarity with cues that predicted or accompanied the traumatic event. According to learning theories, intrusive memories can be seen as a conditioned response to trauma reminders. However, direct laboratory evidence for the link between fear conditionability and intrusive memories is missing. Furthermore, fear conditioning studies have predominantly relied on standardized aversive stimuli (e.g. electric stimulation) that bear little resemblance to typical traumatic events. To investigate the general relationship between fear conditionability and aversive memories, we tested 66 mentally healthy females in a novel conditioned-intrusion paradigm designed to model real-life traumatic experiences. The paradigm included a differential fear conditioning procedure with neutral sounds as conditioned stimuli and short violent film clips as unconditioned stimuli. Subsequent aversive memories were assessed through a memory triggering task (within 30 minutes, in the laboratory) and ambulatory assessment (involuntary aversive memories in the 2 days following the experiment). Skin conductance responses and subjective ratings demonstrated successful differential conditioning indicating that naturalistic aversive film stimuli can be used in a fear conditioning experiment. Furthermore, aversive memories were elicited in response to the conditioned stimuli during the memory triggering task and also occurred in the 2 days following the experiment. Importantly, participants who displayed higher conditionability showed more aversive memories during the memory triggering task and during ambulatory assessment. This suggests that fear conditioning constitutes an important source of persistent aversive memories. Implications for PTSD and its treatment are discussed.

## Introduction

Intrusive recollection of aversive memories is a core symptom of posttraumatic stress disorder (PTSD) [1]–[3]. It mainly consists of images, thoughts, or perceptions that are recollected involuntarily and in a vivid, emotional way, often giving patients the impression that the respective events are happening in the here and now [1]. Intrusive memories are easily triggered by a wide range of stimuli that do not necessarily resemble aspects of the trauma in an obvious meaningful way, but do often have sensory similarity with stimuli that occurred before or during the trauma (e.g., similarities in color, shape, sound, or smell [3]–[6]). These processes are well illustrated by Reemtsma‘s report [7]: he became the victim of a hostage situation and realized afterward that his intrusive memories were triggered by hearing footsteps or a knocking sound. This was caused by the fact that he had heard footsteps approaching the cellar before the kidnappers knocked at the door during his captivity (see also [3]). Thus, due to their temporal contiguity with the trauma, trigger-cues have become proper predictors of the traumatic event.

Ehlers and Clark [3], [6] suggest that strong associative learning acts in concert with other memory processes in generating intrusive memories and the ease with which they are triggered in PTSD. They conclude that stimulus-stimulus as well as stimulus-response associations are particularly strong for traumatic material in PTSD. This makes triggering of intrusive memories and emotional responses by related stimuli more likely, even in the absence of subjective awareness of this connection, which accounts for the patients’ frequently reported impression that intrusions come “out of the blue” [3], [6]. Thus, intrusive memories in PTSD can be regarded as conditioned emotional reactions where triggers constitute conditioned stimuli (CS) which predict an aversive event (unconditioned stimulus; UCS) [8], [9]. Consequently, studying aversive memories in a fear conditioning framework could provide further insight into the underlying mechanisms of intrusive memories in PTSD.

Associative learning processes have been studied extensively to explain the acquisition and maintenance of normal as well as pathological fear, and play a central role in contemporary etiological models of PTSD and other anxiety disorders [9], [10]. The process of extinction has been considered particularly crucial, see e.g. [11], [12], and refers to the gradual decrease in the expression of a conditioned reaction (CR) when a conditioned stimulus (CS) that has previously been coupled with an aversive event (UCS) is presented repeatedly without being followed by a UCS. Rather than being a passive process of erasure or overwriting of the original CS-UCS association, fear extinction is now viewed as an active learning process where an organism learns that a previously threatening stimulus no longer signals danger [13]. Persistent reactivity to a CS even in the absence of a CS-UCS contingency has been shown to be a feature of PTSD [14]–[18] and mirrors the course of the disorder where reactions to trauma-related cues do not decay over time. However, group differences have not only been reported during fear extinction but also during fear acquisition where heightened conditioned responses have been observed in PTSD patients [14], [15]. In summary, these results indicate that PTSD patients show enhanced *conditionability*, that is, a tendency to ‘acquire a *larger* and *more persistent* (autonomic) differential response to an aversive CS’ ( [15]; i.e., higher differential responding during acquisition and/or extinction [10], [19]).

Yet one shortcoming of previous fear conditioning experiments is their relatively poor correspondence with naturally occurring traumatic situations. Typical UCSs – supposedly representing traumatic events in this laboratory analog – include electrical stimulation, aversive noises (e.g., loud white noise or human cries), aversive pictures, or other kinds of basic aversive stimulation such as unpleasant smells or air blasts [10], [20]. Such stimuli only partially depict the typical features (e.g. the dynamic time course) of situations usually involved in fear acquisition. Moreover, they are unlikely to generate the kind of complex memories that could later give rise to intrusive recollection and are thus inappropriate to investigate the relationship between fear conditioning and aversive memories although such a relationship is strongly supported by clinical observations (see above).

Another experimental paradigm that has been intensely researched as a laboratory analog of trauma exposure and subsequent intrusive memories in healthy individuals is the trauma film paradigm. Unlike the brief and simple types of UCSs employed in fear conditioning studies, this research tradition uses film sequences that contain stressful or traumatic events, featuring actual or threatened death or serious injury to the body or self. Following film exposure, diaries are used to assess individuals’ intrusive memories, which are typically weaker but functional analogous to intrusions in PTSD (see [21], for an extensive review). However, the trauma film paradigm is less well suited to experimentally separate potential *triggers of intrusive memories* – as introduced above – from the *intrusive memory content* itself. The dynamic dramaturgy of the film overlays CSs and UCSs in various temporal configurations, thereby precluding the systematic study of conditioning. High temporal precision and clear separation of CSs and UCSs is however afforded by differential fear conditioning paradigms.

Therefore, we combined the two research traditions of fear conditioning and trauma film memory and developed a *conditioned-intrusion paradigm* that can be used to link individual conditionability on the one hand to the individual strength of subsequent aversive memories of the aversive event (UCS) on the other hand. More precisely, short aversive film clips depicting interpersonal violence were included as UCS, aiming to establish – within the bounds of experimental ethics – a reasonably naturalistic *fear conditioning procedure*. Neutral sound clips were either paired with aversive film clips (CS^+^ sound) or were presented unpaired (CS^−^ sound). In contrast to previous fear conditioning tasks with simple and short UCSs, this task has the advantage of generating a more complex memory trace during conditioning which can then be probed subsequently. To assess the time course and potential triggering of aversive memories, the fear conditioning procedure was followed by a *memory triggering task* and an *ambulatory assessment of aversive memories*. The memory triggering task was designed to model plausible conditions of aversive memories in PTSD: it assesses whether the CS^+^ sound, played at subtle volume and embedded in a complex background soundscape, would trigger aversive memories and increase anxiety. Two additional soundscapes, one with the CS^−^ sound embedded and a no-cue condition, served as control conditions. In addition, we assessed aversive memories between day 0 and day 2 after the laboratory session to extend our results to more spontaneous aversive memories in daily life.

We expected to observe successful conditioning in our new fear conditioning task as represented by differential skin conductance responses and subjective ratings (CS valence and fear, UCS expectancy). For the memory triggering task, we expected the highest intensity of aversive memories during the CS^+^ cue condition as compared to the CS^−^ cue or the no-cue condition on subjective (self-reported frequency, duration, and stressfulness of memories, state anxiety) and electrodermal indices. We further predicted that higher conditionability would give rise to stronger aversive memories during the memory triggering task, particularly during the CS^+^ cue condition. A similar relationship was expected for ambulatorily assessed aversive memories. Evidence for such relationships would suggest that individual conditionability contributes to persistent aversive memories, a piece of evidence that has been missing in the literature so far.

## Methods

### Ethics Statement

The study was approved by the ethics committee of the University of Salzburg and written informed consent was obtained from all participants. Before participation, experimental procedures were described in detail, including the presentation of exemplary movie scenes resembling those shown within the study. Participants were furthermore informed that they could indicate to stop the experiment and withdraw from their participation at any time with full compensation.

### Participants

A total of 66 female participants (age: *M* = 23.44, *SD* = 3.69) were recruited at the University of Salzburg, Austria, and participated in exchange for course credit or 25 Euro. The sample comprised only female participants and was fairly large because one aspect of the study will be the investigation of fear conditioning and aversive memories in relation to female gonadal hormones (27.3% of participants used oral hormonal contraception, 31.8% were without hormonal contraception during early follicular phase of their menstrual cycle, and 40.9% were without hormonal contraception during luteal phase). Participants did not report any mental or neurological disorders and were free of medication except for oral hormonal contraceptives. Further exclusion criteria included past experiences of severe interpersonal violence, as well as extensive habitual consumption of TV and film footage or video games including severe violence (consumption exceeding 3 times per week).

Data of an earlier subsample (N = 20) of the current study have been published elsewhere [22]. This preliminary analysis solely covered aspects of the fear conditioning procedure and did not encompass data from the memory triggering task. It did not investigate aversive memories, which is the focus of the current study.

### Procedure

After being welcomed to the laboratory, participants completed several questionnaires, including assessment of trait anxiety (State-Trait Anxiety Inventory, STAI, German version by [23]), depressive symptoms (General Depression Scale, ADS-L; German version by [24]), general medical and psychological health conditions, and consumption of TV and film footage or video games depicting severe violence. Participants were then seated in front of the computer monitor, electrodes for skin conductance measurement were attached, and a saliva sample was collected (data not reported here). Subsequently, participants sat quietly for 2.5 min, allowing them to further adapt to the laboratory environment.

#### Fear conditioning procedure

During a pilot study (N = 9) we had selected 2 neutral sounds with a duration of 5 s each (sound A: sound of a *typewriter*; sound B: *clock ticking*) out of a total of 6 sounds for uses as conditioned stimuli (CS) in the main study. The 2 sounds were both naturalistic, matched on valence and arousal and could plausibly be paired with our unconditioned stimuli (UCSs). Three film scenes (each with a duration of 25 s) were extracted from commercial movies to be used as UCS [22] (film A: scene from “Antichrist”, 2009, directed by Lars von Trier; film B: scene from “Hostel”, 2005, directed by Eli Roth; film C: scene from “Scar”, 2007, directed by Jed Weintrob). Each film scene depicted severe violence between 2 persons or the moment immediately after such an attack, showing a severely injured person. The pilot study data verified that the selected film scenes were matched with respect to valence and arousal.

CS sounds were assigned to CS^+^ (i.e., sound that was later followed by an aversive film clip, thus representing a danger signal) and CS^−^ (i.e., sound that was never followed by an aversive film clip, thereby representing a safety signal), counterbalanced over participants (for a closer description of mechanisms involved in the responding to CS^+^ and CS^−^ see e.g., [25]). (See File see *Figure S1 Supporting information about effects of assignment of sounds to CS^+^/CS* ^−^ for analyses on the degree of associability of the CS sounds with the violent film clips. In short, no consistent pattern in favor of one of the sounds emerged.).

**Figure 1:**
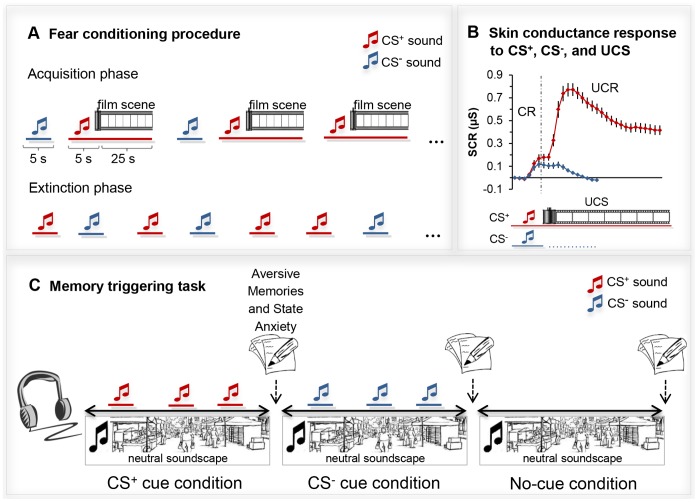
Schematic depiction of the conditioned-intrusion paradigm. *Panel A:* Differential fear conditioning procedure with aversive film scenes as unconditioned stimuli (UCS) and sounds as conditioned stimuli (CS^+^ and CS^−^). See text for details. *Panel B:* Red line: Average skin conductance response to the CS^+^ and the UCS during the reinforced trials of the acquisition period. Blue line: Average skin conductance response to the CS^−^ during acquisition. The dashed line displays the boundary between CS and UCS presentation. Values are referenced to the respective baseline before CS onset; means and standard errors are displayed. *Panel C:* Memory triggering task. Neutral soundscape with superimposed CS^+^ cues (CS^+^ cue condition), CS^−^ cues (CS^−^ cue condition), or no superimposed sound cues (no-cue condition). See text for details.

The conditioning procedure consisted of a habituation, an acquisition, and an extinction phase. Prior to habituation, written instructions informed participants that they are going to hear the sound of a typewriter and of a clock and that there are no aversive film clips going to be displayed during this phase of the experiment. During the habituation phase, CS^+^ and CS^−^ sounds were presented 6 times for a duration of 5 s each, in pseudorandom order. Throughout the task, an intertrial interval of 12–20 s (determined pseudo-randomly) was used. After the habituation phase, participants were informed that the 2 sounds would be presented again in the next part of the experiment and that one of the sounds could now be followed by a film scene, whereas the other sound would not be followed by film scenes (similar instructions for contingencies between CS and UCS are common in fear conditioning research and have previously been used e.g., by [26] and [14]). The acquisition phase consisted of 8 CS^+^ sounds of 5 s duration, of which 6 were followed by an aversive film clip (each of 3 film clips presented 2 times in pseudorandom order, 75% reinforcement). During the film clips the CS^+^ sound kept playing at lowered volume in the background. The acquisition phase further comprised 6 CS^−^ sound presentations of 5 s duration, not followed by a film clip. During the extinction phase, the CS^+^ and CS^−^ were both presented 6 times with the CS^+^ sound no longer followed by a film clip. The fear conditioning procedure and the subsequent memory triggering task are schematically depicted in *Figure 1* (*panels A* and *C*).

Several online ratings were collected during the conditioning procedure using on-screen visual analog scales: CS valence (“How did you experience the sound during its last presentation?” anchors: 0 = very pleasant, 100 = very unpleasant ), fear elicited by the CS sounds (“How much did the sound elicit fear in you during its last presentation?” anchors: 0 = not at all, 100 = very strongly), and expectancy of a UCS (“How much did you expect the sound to be followed by an aversive film clip during its last presentation?” anchors: 0 = very low expectancy, 100 = very high expectancy) were rated at the end of the habituation phase as well as in the middle and at the end of the acquisition and extinction phases. The UCS expectancy rating was phrased slightly differently when being displayed at the end of habituation (“How much do you expect this sound to be followed by an aversive film clip during the subsequent experiment?”) as compared to the rest of the procedure. Thus, post-habituation UCS expectancy ratings do not represent the degree to which participants expected an aversive film during habituation, but rather the extent to which the sounds were *à priori* associated with the appearance of an aversive film clip.

At the end of the conditioning procedure participants rated valence separately for each film clip (visual analog scale; 0 = very pleasant, 100 = very unpleasant). Furthermore, contingency awareness (CA) was assessed at the end of the conditioning procedure by asking for each CS-type whether they were paired with the UCS. Only participants who correctly reported CS^+^ as being paired and CS^−^ as being unpaired with aversive film clips were scored as contingency aware. However, note that a systematic investigation of effects of CA on fear conditioning and triggering of aversive memories goes beyond the scope of the current study and requires specific adaptations of experimental procedures. Data from all participants are reported in the results section whereas preliminary data on the role of CA can be found in *Figure S2 Supporting information about effects of contingency awareness*.

#### Memory triggering task

After a ∼30 min break used to complete questionnaires and to collect another saliva sample, the memory triggering task commenced. This task was designed to model daily life situations in which trauma survivors might experience intrusive memories and to investigate the potential of CS sound cues to trigger such memories. Written instructions informed participants that they would now be presented with a background soundscape via earphones while they could let their mind wander freely. The task consisted of 3 almost identical soundscapes of 3 min duration, each being preceded by a 20 s silent period allowing participants to recover between conditions. The basic soundscape featured various people talking with neither content nor language identifiable for participants, as may occur at a marketplace or shopping mall. In the *CS^+^ cue condition* the CS^+^ sound (typewriter or clock ticking) was superimposed 6 times with 7 s duration on the soundscape audio track. Similarly, in the *CS* ^−^ *cue condition*, the CS^−^ sound was superimposed 6 times with 7 s duration. In the *no-cue condition* there were no sound cues superimposed. Both in the CS^+^ and the CS^−^ cue conditions, sound cues were superimposed subtly but clearly perceptible, at the same time points (2 in minute 1, 2 in minute 2, 2 in minute 3). Superimposing CS sound cues on a neutral, daily life background soundscape (i.e., the marketplace/shopping mall soundscape) allowed for displaying CS sound cues unobtrusively in a controlled but naturalistic context. The order of conditions was counterbalanced (including all 6 permutations of conditions) across participants.

Subsequent to each 3 min soundscape, participants coded their response to the previous condition on the STAI state questionnaire [23] and the Intrusion Memory Questionnaire (IMQ) [27], [28]. The IMQ was adapted to assess number and duration of memories (% of the total time) as well as distress (visual analog scale with the anchors “0 = not distressing at all” and “100 = extremely distressing”) elicited by memories experienced during the previous soundscape/condition. IMQ instructions defined memories as “images or thoughts about the film scenes” but also the “presence of thoughts or feelings” that participants had when watching the film clips. Importantly, participants were told to solely count memories that “came up spontaneously” and not through deliberate recall to capture memories resembling intrusions in PTSD as closely as possible [27], [28]. To reduce alpha inflation in subsequent correlation analyses (see below) and to obtain a more reliable score of aversive memories, we additionally calculated an index of aversive memories by building a sum score for the IMQ questionnaire separately for each condition of the memory triggering task by standardizing and summing single items. (Single item responses were scaled differently and thus needed to be transformed into *z*-scores taking all subjects and points of measurement into account. For purposes of better illustration within figures and tables, *z*-scores were further transformed into *T*-scores.) Cronbach alphas ranged between.862<*α*<.915, pointing to the feasibility of this approach.

#### Ambulatory assessment of aversive memories

To extend our analyses from short-term aversive memories in the laboratory to involuntary retrieval of aversive memories in daily life conditions, participants completed ambulatory assessments the day of the experiment and on the 2 following days. Because theoretical considerations and a pilot study indicated strong reactivity effects when participants were asked to protocol each aversive memory immediately after its occurrence, we did not collect continuous electronic diary data [29]. Instead, participants completed the adapted version of the IMQ before going to bed on the experiment day (day 0) as well as on day 1 and day 2 post-experiment, allowing us to assess the course of aversive memories over a few days. Definition of memories was identical to that used during the memory triggering task, with the only difference being that participants should now also count memories of sounds that they had encountered during the experiment. Again, standardized sum scores were calculated for memories assessed by the IMQ on day 0 to day 2 and these sum scores were furthermore averaged over days to get a more reliable mean score of aversive memories for subsequent correlation analyses (Cronbach alpha:.876). On day 2, participants furthermore completed the Impact of Event Scale – revised (IES-R; German version by [30]) measuring intrusion, avoidance, and hyperarousal related to the film clips retrospectively for day 0 until day 2 after the laboratory session.

Finally, participants were orally debriefed and reimbursed for study participation. Particular emphasis was given to the possibility of contacting the experimenters in case of further questions or distress due to the experiment.

### Apparatus and Physiological Recordings

During the laboratory session, participants were seated on a chair placed 50 cm in front of a 24″ full-HD monitor. Stimulus presentation and behavioral data acquisition were controlled by E-Prime 2.0 (Psychology Software Tools, Inc., Pittsburgh, PA, USA). Acoustic stimuli were presented via shielded earphones at a constant volume across participants. Skin conductance (SC) was measured using Ag/AgCl electrodes filled with isotonic electrode paste [31]. Electrodes were placed on the middle phalanx of the index and middle fingers of the non-dominant hand. Recording of SC data was performed with a sampling rate of 1000 Hz using the software Polybench 1.22 (TMSi, Twente Medical Systems International, EJ Oldenzaal, Netherlands), a Porti 32-channels-amplifier (TMSi), and an SC-amplifier (Becker Meditec, Karlsruhe, Germany). Further analyses of SC data were conducted using ANSLAB 2.51 [32]. (Note that we refer to skin conductance in general as SC, whereas SCL specifically means skin conductance level (i.e., the tonic level of skin conductance) and SCR specifically indicates skin conductance response (i.e., phasic alterations in skin conductance elicited by stimuli) [33]).

### Data Reduction and Statistical Analyses

A deficient unconditioned reaction (UCR) during the fear conditioning procedure was used as an exclusion criterion for all further analyses, since this could either imply measurement problems or insufficient aversiveness of film clips for a minority of participants. A mean UCR <0.2 µS was used as exclusion criterion with UCR calculated by subtracting mean baseline skin conductance level (SCL, −1 to 1 s relative to film clip onset, taking an SCR onset delay into account) from the maximum SCL during the remaining 24 s of the film clip, considering only the first presentation of each film clip. (Note that SCRs show a typical waveform characteristic that has been well described, including an onset delay of 1–1.5 s after stimulus presentation [33]. By shifting the SCR analysis window for UCR by 1 s (see e.g., [17]), we took this SCR response delay into account to separate CR und UCR.) Two participants (3.0%) were excluded based on the UCR exclusion criterion.

For the conditioning procedure, we calculated a skin conductance response (SCR) by subtracting the average pre-CS baseline SCL (−2 to 0 s relative to CS onset) from the maximum CS SCL (0 to 6 s relative to CS onset, again taking an SCR response delay into account) and normalized SCR data using the natural logarithm of 1+SCR (in µS). Responses to each CS-type on 3 consecutive presentations were then averaged resulting in 2 blocks per conditioning phase.

For the memory triggering task, mean SCL for each condition was calculated as the average across the whole phase (3 min) of each condition and SCL data were again normalized using the natural logarithm of 1+SCL (in µS).

#### Statistical analyses of fear conditioning procedure

For ratings of CS valence, CS fear, and UCS expectancy, a MANOVA including CS-type (CS^+^, CS^−^) and Time (first and second half of each phase) as within-participant factors was calculated for each conditioning phase (habituation, acquisition, and extinction) (with Pillai’s trace as a test statistic). Since only one measurement point existed for ratings during habituation, the MANOVA for habituation did not include the factor Time. Significant MANOVAs were followed by repeated measures ANOVAs (or *t*-tests for habituation) for each behavioral rating in order to reveal effects of CS-type and Time on specific rating scales. For SCRs, we calculated repeated measures ANOVAs separately for each conditioning phase, again including CS-type (CS^+^, CS^−^) and Time (first and second half of each phase) as within-participant factors. (Only CS^+^ presentations that were reinforced by an aversive film clip were included in the SCR analysis. However, an analysis for the acquisition phase that also included unreinforced CS^+^ showed that their inclusion does not essentially alter results.).

#### Statistical analyses of memory triggering task

Repeated measures ANOVAs were calculated with the within-participant factor Condition (CS^+^ cue condition, CS^−^ cue condition, no-cue condition) for each outcome measure IMQ, STAI state anxiety, and SCL.

The alpha level for all analyses was set to.05 and significant main or interaction effects of ANOVAs were further explored using *t-*tests. For all MANOVAs, ANOVAs and *t*-tests, effect sizes are reported (partial eta squared *η^2^* or Cohen’s *d*, respectively). When the sphericity assumption was violated in ANOVAs, the Greenhouse-Geisser correction for repeated measures was applied with nominal degrees of freedom being reported.

#### Statistical analyses of correlations between fear conditionability and aversive memories

A score for *fear conditionability* was calculated for each outcome variable, indexing the degree of previously acquired and non-extinguished fear responding. More precisely, this index measures the extent to which previously acquired fear responding remains above habituation response levels during extinction: for each measure (CS valence, fear, UCS expectancy, SCR) and CS-type (CS^+^, CS^−^), end of habituation values served as individual pre-conditioning baselines and were subtracted from average extinction values.(Note that subtracting habituation response levels was crucial since we noted substantial interindividual differences in habituation phase responding. Although there was no mean difference between ratings of CS^+^ and CS^−^ at the end of habituation, a closer examination of the data revealed that participants differed considerably in their responses to one or the other CS. This is probably due to the fact that we chose complex, everyday life sounds that may bring about a wide range of idiosyncratic associations and appraisals. Subtracting habituation response levels ensures that individual *à priori* (habituation) responding is separated from conditioned responding, thereby preventing an over- or underestimation of individual conditionability. A similar approach has recently been taken by Dunsmoor et al. [34].) A differential conditionability index was then computed on these “baseline-referenced” scores for each individual by subtracting the CS^−^ score from the CS^+^ score. Higher scores on this index represent stronger and more persistent responding to the CS^+^ relative to the CS^−^ and thus heightened *conditionability* as defined by Orr et al. [15].

Conditionability scores for each outcome variable were then correlated with the IMQ sum scores during the memory triggering task, separately for the CS^+^ cue, the CS^−^ cue, and the no-cue condition (Bonferroni corrected in the self-report and the autonomic domain (9 and 3 correlations, respectively)). Similarly, scores for each outcome variable were correlated with the averaged IMQ sum score for day 0 until day 2 after the laboratory session (Bonferroni corrected for 3 self-report correlations). Spearman’s correlation coefficients (*Spearman’s rho*, *ρ*) were calculated due to non-normal bivariate distributions. All statistical analyses were performed using PASW Statistics 18 (SPSS Inc., Chicago, IL, USA).

## Results

### Demographic and Psychometric Variables

Seven participants had to be excluded from the analyses due to early termination of the study (3 participants), excessive consumption of films or video games including severe violence (1 participant, see definition above), insufficient unconditioned responding (2 participants, criteria explained above) or experimenter error (1 participant), reducing the final sample to 59 participants (age: *M* = 23.37 years, *SD* = 3.44). Trait anxiety and depressive symptoms were in the normal range (trait anxiety: *M* = 37.97, *SD* = 9.58, [23]; depressive symptoms: *M* = 12.57, *SD* = 7.75, [24]). Concerning consumption of TV and film footage or video games depicting severe violence, 67.8% reported consumption of such material between almost never and a few times per year, 20.3% between 1 and 3 times per month, and 11.9% between 1 and 3 times per week.

### Fear conditioning procedure

#### Ratings of CS valence, CS fear and UCS expectancy

**Figure 2:**
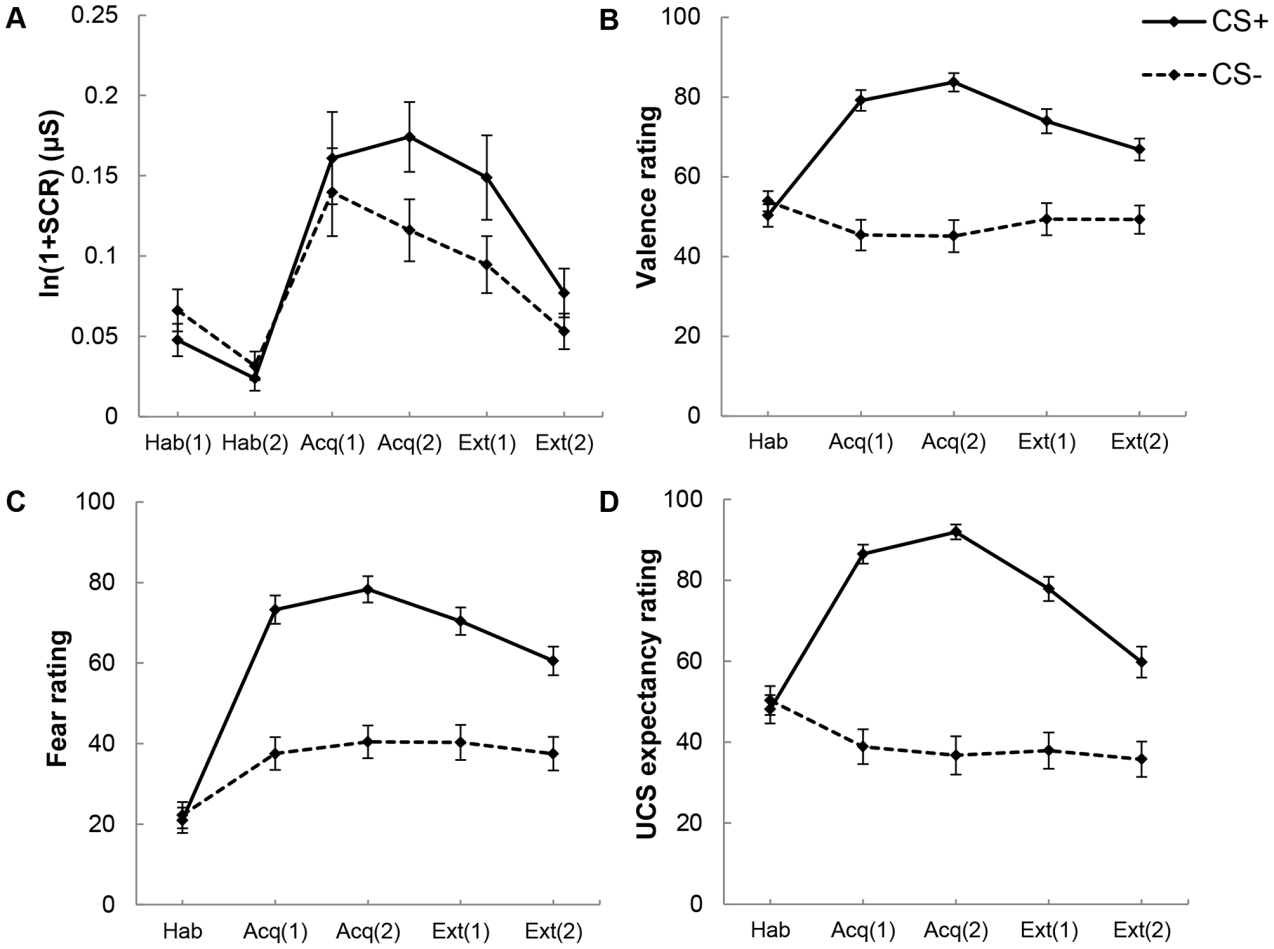
Physiological and behavioral results of the fear conditioning task. Means and standard errors of skin conductance response (SCR, *panel A*), valence ratings (*panel B*), fear ratings (*panel C*), and unconditioned stimulus (UCS) expectancy ratings (*panel D*) in response to CS^+^ and CS^−^ across habituation (Hab), acquisition (Acq), and extinction (Ext) phases. See text for details.

The MANOVA for behavioral ratings during habituation showed no significant effect of CS-type, *F*(3,56) = 0.39, *p* = .764, indicating that CS^+^ and CS^−^ did not differ on ratings of CS valence, CS fear, or UCS expectancy at the end of the habituation phase. For acquisition, the MANOVA displayed a main effect of CS-type, *F*(3,56) = 41.16, *p*<.001, *η^2^* = .69, whereas there was no significant effect of Time, *F*(3,56) = 1.81, *p* = .156, nor a CS-type x Time interaction, *F*(3,56) = 1.01, *p* = .396. (Note that statistical parameters of follow-up ANOVAs are displayed in *Table 1*. ANOVA main effects of Time are only reported in the text if interacting with CS-type.) Separate, measure-wise follow-up ANOVAs for ratings during acquisition displayed a CS-type effect on each single rating scale, due to higher ratings on negative valence, fear, and UCS expectancy for the CS^+^ compared to the CS^−^. During extinction, the MANOVA displayed a main effect of CS-type, *F*(3,56) = 20.34, *p*<.001, *η^2^* = .52, as well as a main effect of Time, *F*(3,56) = 6.58, *p* = .001, *η^2^* = .26, which were modulated by a significant CS-type x Time interaction, *F* = (3,56) = 7.43, *p*<.001, *η^2^* = .29. Separate follow-up ANOVAs confirmed CS-type main effects, modulated by CS-type x Time interactions for each rating scale during extinction. The CS^+^ was rated more negatively and elicited more fear and UCS expectancy during mid-extinction, all *t*s(58)>5.18, *p*<.001, *d*>0.90, and end of extinction, all *t*s(58)>4.80, *p*<.001, *d*>0.72. However, the interaction effect pointed to a decrease in negative valence, fear and UCS expectancy for the CS^+^, all *t*s(58)>3.87, *p*<.001, *d*>0.32, as opposed to stable levels for the CS^−^, all *t*s(58)<1.16, *p*>.251. *Figure 2* (*panel B–D*) displays means and standard errors for ratings of CS^+^ and CS^−^.

**Table 1:** ANOVA effects of the fear conditioning procedure for valence, fear, and unconditioned stimulus (UCS) expectancy ratings as well as skin conductance response (SCR).

	ANOVA		
	CS-type, *F, p, η^2a^*	Time, *F, p, η^2a^*	CS-type x Time, *F, p, η^2a^*
***Valence***			
Acquisition	75.57, <.001*, .57	1.73, .193, n.s.	1.48, .229, n.s.
Extinction	27.56, <.001*, .33	7.74, .007*, .12	6.63, .013*, .10
***Fear***			
Acquisition	74.99, <.001*, .56	5.60, .021*, .09	0.46, .498, n.s.
Extinction	47.02, <.001*, .45	12.67, .001*, .18	7.33, .009*, .11
***UCS expectancy***			
Acquisition	125.96, <.001*, .69	1.15, .289, n.s.	2.34, .132, n.s.
Extinction	59.14, <.001*, .51	20.28, <.001*, .26	22.64, <.001*, .28
***SCR***			
Habituation	4.09, .048*, .07	21.70, <.001*, .27	0.46, .500, n.s.
Acquisition	10.86,.002*, .16	0.14, .712, n.s.	3.12, .082, n.s.
Extinction	16.95, <.001*, .23	17.23, <.001*, .23	4.22, .044*, .07

*Note:* *significant at p<.05; n.s. = not significant. Note that the MANOVA for behavioral ratings during habituation did not display a significant main effect of CS-type and was thus not followed up by analyses for each single rating scale (see text for details).

a *F*(1,58) for factor CS-type, factor Time, and for interaction effect CS-type x Time.

#### Skin conductance response

*Figure 1* (*panel B*) illustrates the conditioned and unconditioned responses as measured by skin conductance during the acquisition period in second-by-second plots. Mean SCR values for both CSs and all phases are depicted in *Figure 2* (*panel A*) and statistical parameters of ANOVAs for SCR are given in *Table 1*. During habituation, there was a marginally significant main effect of CS-type. The CS^−^ elicited a slightly higher SCR than the CS^+^. (Note that this main effect was not due to unequal counterbalancing of typewriter/clock to CS^+^/CS^−^: assignments were evenly split. Furthermore, there was no effect of assignment of sounds (typewriter/clock) to CS^+^/CS^−^ on SCR during habituation, *p*s>.117.) For acquisition, there was a main effect of CS-type indicating that the CS^+^ elicited a higher SCR than the CS^−^. During extinction, a CS-type main effect was modulated by a CS-type x Time interaction. Whereas the CS^+^ elicited a higher SCR than the CS^−^ during the first half of the extinction period, *t*(58) = 4.55, *p*<.001, *d* = 0.32, this difference was reduced to trend level during the second half of extinction, *t*(58) = 1.97, *p* = .054.

#### Film clip valence

Mean valence of film clips was 87.62 (SD = 11.28; 0 = very pleasant, 100 = very unpleasant) with valence of the 3 film clips not significantly differing from each other, *F*(2,116) = 1.52, *p* = .222. Thus, films were experienced as fairly unpleasant.

#### Contingency awareness

The percentage of participants who failed to correctly report contingencies after extinction was 18.6% (see *Figure S2 Supporting information about effects of contingency awareness* for analyses including CA as a between-group factor).

### Memory Triggering Task

#### Aversive memories (IMQ questionnaire data) and state anxiety

As expected, responses to the IMQ (including subjective aversive memory frequency and duration as well as distress through aversive memories) and STAI state anxiety differed significantly by condition of the memory triggering task which comprised neutral soundscapes with CS^+^, CS^−^, or no superimposed sound cues. Respective means and standard deviations as well as inferential statistics for single items of the IMQ, IMQ sum score and other outcome variables of the memory triggering task are displayed in *Table 2*. During the CS^+^ cue condition, participants reported more, longer, and more distressing memories, all *t*s(58)>2.94, *p*<.005, *d*>0.40, as well as more state anxiety, *t*(57) = 3.37, *p* = .001, *d* = 0.26, than during the CS^−^ cue condition. The CS^−^ cue condition, in turn, triggered more, longer and more distressing memories, all *t*s(58)>2.93, *p*<.005, *d*>0.27, as well as stronger state anxiety, *t*(57) = 2.34, *p* = .023, *d* = 0.20, than the no-cue condition. (Note that one participant accidentally forgot to complete one STAI state questionnaire during the memory triggering task, which led to reduced degrees of freedom.).

**Table 2:** Results for aversive memories, state anxiety, and SCL during the memory triggering task.

	Memory triggering task			
	CS^+^ cue cond.	CS^−^ cue cond.	No-cue cond.	Inferential statistics
	*M (SD)*	*M (SD)*	*M (SD)*	*F, p, η^2^*
IMQ – Sum score^ab^	62.04 (28.64)^1^	48.7 (26.61)^2^	39.25 (21.80)^3^	29.29, <.001*, .34
IMQ – Number^b^	4.36 (3.78)^1^	2.71 (3.02)^2^	1.62 (2.01)^3^	24.49, <.001*, .30
IMQ – Duration^b^	35.29 (28.33)^1^	24.69 (23.97)^2^	15.86 (20.63)^3^	19.62, <.001*, .25
IMQ – Distress^b^	41.69 (28.17)^1^	29.46 (30.04)^2^	21.61 (28.70)^3^	22.60, <.001*, .28
State anxiety^c^	46.39 (12.42)^1^	43.26 (11.73)^2^	40.9 (10.96)^3^	17.28, <.001*, .23
SCL^c^	1.864 (.305)^1^	1.855 (.312)	1.848 (.305)^2^	3.55,.032*, .06

*Note: cond.*: condition; *IMQ number*: number of aversive memories (“How often did memories (pictures or thoughts) of violence scenes pop into your mind during the last part of the experiment (the last 3 minutes)?”); *IMQ duration*: duration of aversive memories in % of total time of the respective condition (“How many percent of the time have you been mentally engaged with memories (pictures or thoughts) of the violence scenes during the last part of the experiment (the last 3 minutes)?”); *IMQ distress*: distress elicited by aversive memories, scale 0–100; 0 = not distressing at all, 100 = extremely distressing (“How distressing did you find these memories of the violence scenes during the last part of the experiment (the last 3 minutes)?”); *state anxiety*: assessed by STAI state scale; *SCL*: skin conductance level given as ln(1+SCL) in µS.

* significant main effect of condition at *p*<.05.

1 2 3 Different number superscripts indicate that conditions significantly differ from each other at *p*<.05 in post-hoc tests.

a Values represent sum scores of the IMQ in T-scores.

b F(2,116).

c F(2,114).

#### Skin conductance level

Due to problems with skin conductance recording during the memory triggering task, one participant had to be removed from analyses of SCL for that task. As expected, SCL significantly differed by condition (type of cue overlaid over the soundscape). *Post-hoc* tests revealed that SCL was not different for the CS^+^ cue condition as compared to the CS^−^ cue condition, *t*(57) = 1.43, *p* = .157. However, SCL during the CS^+^ cue condition was higher than during the no-cue condition, *t*(57) = 2.64, *p* = .011, *d* = 0.05, whereas this was not the case for the CS^−^ cue condition, *t*(57) = 1.23, *p* = .225.

### Correlations between Fear Conditionability and Aversive Memories during Memory Triggering Task

**Figure 3:**
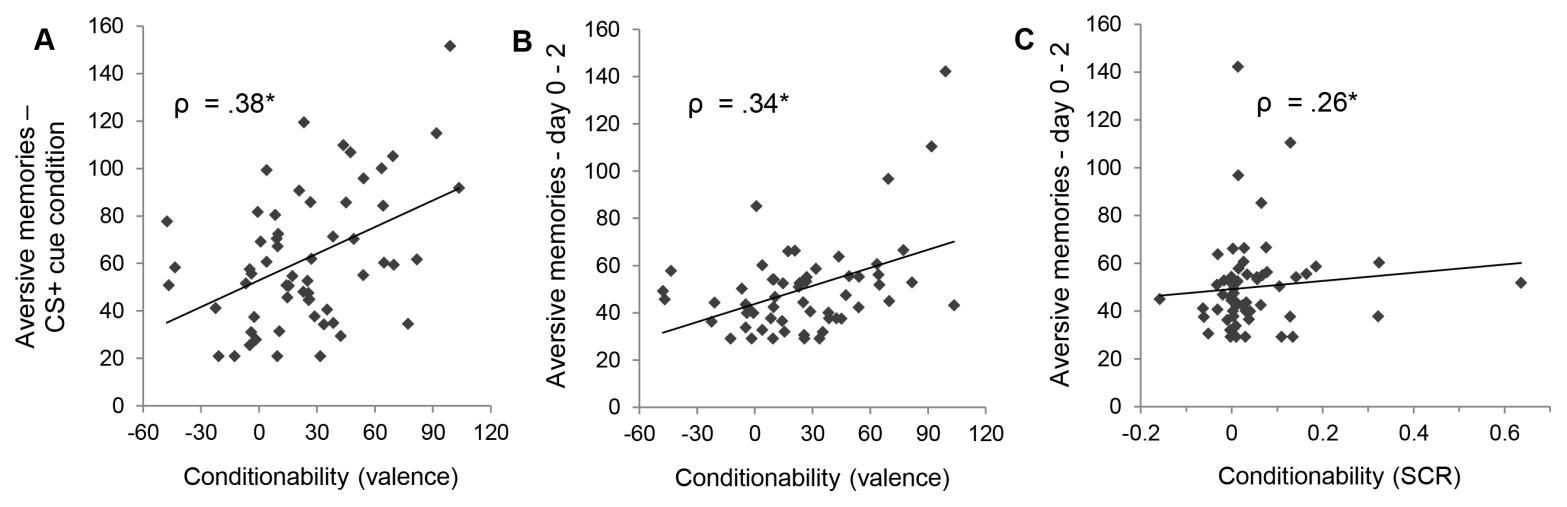
Correlations between fear conditionability and aversive memories. *Panel A*: Correlation between conditionability as measured by valence and aversive memories during CS^+^ cue condition of the memory triggering task. *Panel B*: Correlation between conditionability as measured by valence and ambulatorily assessed aversive memories. *Panel C*: Correlation between conditionability as measured by skin conductance response (SCR) and ambulatorily assessed aversive memories. Note: Values for aversive memories represent sum scores of the Intrusion Memory Questionnaire (IMQ) in T-scores. Variables did not fulfill bivariate normal distribution criteria and thus non-parametric correlational analyses were used. See text for details.

A significant correlation was found between aversive memories during the CS^+^ cue condition and conditionability as measured by valence ratings (*ρ* = .38, *p* = .003; Bonferroni adjusted alpha level used as significance threshold:.05/9 = .006) (see *Figure 3*, *panel A*). Participants whose acquired differential negative evaluations remained relatively high during extinction, reported stronger (i.e., more, longer and more distressing) aversive memories during the CS^+^ cue condition. None of the other scores for fear conditionability (based on SCR or ratings of CS fear or UCS expectancy) were significantly correlated with aversive memories during the CS^+^ cue condition (all *p*s>.229). Furthermore, as expected, no significant correlations emerged between fear conditionability and aversive memories during the CS^−^ and no-cue condition. *Table 3* lists all correlations between scores for fear conditionability and aversive memories during the memory triggering task by condition.

**Table 3:** Correlations between fear conditionability and aversive memories (as assessed by the IMQ) during the memory triggering task and ambulatory assessment.

	Memory triggering task (IMQ)			Ambulatory assessment (IMQ)
Fear conditionability	CS^+^ cue cond.	CS^−^ cue cond.	No-cue cond.	Day 0 to day 2
	*ρ (p*)	*ρ (p*)	*ρ (p*)	*ρ* (*p*)
Valence	.38* (**.003**)	.22 (.091)	.14 (.282)	.34* (**.009**)
Fear	.09 (.512)	−.14 (.283)	−.13 (.339)	.02 (.893)
UCS expectancy	.16 (.229)	−.05 (.683)	−.03 (.819)	.02 (.891)
SCR	.15 (.259)	.23 (.078)	.14 (.278)	.26* (**.048**)

*Note: cond.*: condition; *IMQ*: Intrusion Memory Questionnaire.

Non-parametric correlation coefficients (Spearman’s rho, *ρ*) are reported. See text for details.

### Ambulatory Assessment of Aversive Memories

Means and standard deviations for ambulatorily assessed aversive memories as measured by the IMQ between days 0 and 2 are displayed in *Table 4*. Furthermore, *Table 4* displays the sum scores for intrusion, avoidance, and hyperarousal as assessed by the IES-R on day 2 (retrospective for days 0–2) after participation in the laboratory session.

**Table 4:** Ambulatory assessment of aversive memories.

	Ambulatory assessment		
	Day 0	Day 1	Day 2
	*M (SD)*	*M (SD)*	*M (SD)*
**IMQ**			
Number	4.34 (5.24)	2.17 (3.70)	1.27 (2.28)
Total duration (in min)	12.27 (14.85)	5.03 (7.86)	1.89 (3.08)
Distress (0–100)^a^	37.63 (28.37)	20.00 (24.84)	11.36 (19.52)
**IES-R** b			
Intrusion	–	–	7.85 (6.48)
Avoidance	–	–	9.05 (7.25)
Hyperarousal	–	–	2.90 (4.77)

*Note: IMQ*: Intrusion Memory Questionnaire; *IES-R:* Impact of Event Scale – revised.

a 0 = not distressing at all, 100 = extremely distressing.

b retrospective for day 0 until day 2; possible scores: intrusion 0–35, avoidance 0–40, hyperarousal 0–35.

### Correlations between Fear Conditionability and Ambulatorily Assessed Aversive Memories

No correlations were found between fear conditionability as measured by fear or UCS expectancy ratings and ambulatorily assessed aversive memories (all *p*s>.891). However, ambulatorily assessed memories significantly correlated with conditionability as measured by valence (*ρ* = .34, *p* = .009, Bonferroni adjusted alpha level used as significance threshold:.05/3 = .016) (see *Figure 3*, *panel B*), showing that participants whose acquired differential negative evaluations remained higher during extinction, were more prone to report aversive memories on day 0 to day 2 after the laboratory session. Moreover, there was a marginally significant correlation between fear conditionability as measured by SCR and ambulatorily assessed aversive memories (*ρ* = .26, *p* = .048) (see *Figure 3*, *panel C*) – which, however, based on the asymmetrical and dispersed nature of the bivariate distribution, appears not to be particularly robust and needs to be interpreted with caution. The correlation does however indicate that participants with still stronger acquired differential SCRs during the extinction period were reporting stronger aversive memories on day 0 to day 2. *Table 3* lists all correlations between different scores of fear conditionability and ambulatorily assessed aversive memories.

## Discussion

Using a naturalistic fear conditioning paradigm in a healthy sample, the present study revealed that fear conditionability contributes to subsequent aversive memories in the laboratory and in daily life. This finding supports the assumption that intrusive, aversive memories constitute a non-extinguished conditioned emotional reaction to trauma reminders (see e.g., [8], [9]). From a methodological point of view our results demonstrate successful differential fear acquisition and extinction in a novel fear conditioning task featuring sound CSs and aversive film UCSs, and the potential of such film UCSs to generate aversive memories that can be elicited in a memory triggering task. Thus, our *conditioned-intrusion paradigm* opens up new avenues to study aversive memories within a fear conditioning framework which could be a promising approach to deepen our understanding of the disturbing nature of intrusions in PTSD. In the following we summarize and discuss our results in detail.

During the fear conditioning procedure, participants exhibited differential fear acquisition as indicated by SCR as well as online ratings (CS valence, CS fear, and UCS expectancy), pointing to the power of aversive films as UCSs in fear conditioning. Moreover, film-based UCSs are probably closer to real-life aversive experiences [35], [36] than traditional UCS such as electric shock or aversive noise. In recent years there was a trend towards more ecological validity in fear conditioning – as, for example, illustrated by studies using virtual reality to model different conditioning contexts [37], [38] or studies creating a UCS by combining static visual stimuli with unpleasant sounds [39], [40]. Still, the UCSs implemented in these studies only partially capture the complexity and multisensory nature of real-life aversive experiences and are therefore not optimally suited to elicit aversive memories that can be studied subsequent to a fear conditioning procedure.

During our memory triggering task, however, we could show that aversive film clips are suitable to induce such aversive memories – this is also supported by research building on the trauma film paradigm (see [21] for review). In line with our hypothesis, aversive memories and state anxiety were highest when CS^+^ sound cues were superimposed on a neutral soundscape (CS^+^ cue condition), as compared to the same neutral soundscape when CS^−^ sound cues (CS^−^ cue condition) or no additional sound cues (no-cue condition) were superimposed. Furthermore, SCL was significantly higher during the CS^+^ cue condition as compared to the no-cue condition, whereas SCL during the CS^−^ cue condition did not differ from the other conditions. Hence, the blending of acoustic, conditioned “trauma reminders” into a neutral soundscape apparently led to increased SCL. This cannot solely be explained by an orienting response to sound cues, since interspersion of CS^−^ cues did not result in an equivalent response. Rather, our result indicates an increase in general physiological arousal in the CS^+^ cue condition driven by the sympathetic nervous system [33] (for an overview on psychophysiological responses to trauma reminders, see [41]).

However, although aversive memories and state anxiety were highest during the CS^+^ cue condition of the memory triggering task, they were also elevated during the CS^−^ cue relative to the no-cue condition, even though the CS^−^ was never coupled with an aversive film clip. Albeit we had no clear *à priori* hypothesis concerning potential differences between the CS^−^ cue and the no-cue condition, there are several factors that can account for this finding. First, the CS^−^ might have acquired negative properties due to its presentation in the same acquisition context as the CS^+^ (for a discussion of different context effects in fear conditioning see e.g., [13], [42]). Second, the results can be interpreted in the light of fear generalization. In most differential fear conditioning studies the CS^−^, which can also be seen as a kind of safety cue, is usually included as a control condition with which to contrast conditioned fear to the CS^+^. However, conditioned fear can generalize from CS^+^ to CS^−^. This is the more likely the more the stimuli resemble each other [43] and has been shown to be more pronounced in patients with anxiety disorders [44]–[46]. Such a generalization of aversive experience from CS^+^ to CS^−^ cue conditions might furthermore illustrate how triggers of aversive memories generalize from stimuli that were directly associated with the trauma to stimuli only loosely resembling stimuli encountered during the trauma (e.g., ambient sounds sharing some characteristics with the typewriter or clock ticking, referring to our experiment). A third and related explanation for the observation of elevated response during the CS^−^ cue condition could possibly relate to contingency learning – confusions about UCS prediction during the memory triggering task could potentially promote elevated responses to the CS^−^.

In general, our memory triggering task was designed with the aim of modeling conditions in which PTSD patients might experience aversive memories. However, in conditioning terms, this phase could also be regarded as an extinction recall phase, with altered sensory context. Extinction recall can be described as the retrieval and expression of learned extinction memory when conditioned stimuli are presented again after a delay, and has been shown to be deficient in patients with PTSD [47], [48]. However, we chose a comparatively short delay between the extinction phase and the subsequent memory triggering task (30 minutes in our study as compared to extinction recall on day 2 in [47] and [48]) during which subjects were engaged in a standardized task. Furthermore, we assessed subjects’ aversive memories during the presentation of a neutral soundscape that either included CS^+^, CS^−^ or no additional sound cues, which further distinguishes our memory triggering task from a standard extinction recall phase. Following such an approach, we tried to create an experimental condition most likely fulfilling both the criteria of internal validity as well as external validity with respect to conditions triggering intrusions in PTSD.

As hypothesized, aversive memories during the CS^+^ cue condition of the memory triggering task as well as during ambulatory assessment were partially predicted by our index of conditionability: participants whose acquired differential negative evaluations remained relatively high during extinction reported stronger aversive memories during the CS^+^ cue condition of the memory triggering task as well as in daily life. The change in valence of a stimulus that is due to the pairing of that stimulus with another negative (or positive) stimulus is termed evaluative conditioning (EC) [49], [50] and can occur in parallel with fear conditioning (see e.g., [51]). Whereas earlier models of EC had claimed that evaluatively conditioned effects remain relatively stable in long-term memory once they have been formed [52], [53], a recent meta-analysis by Hofmann et al. [50] concluded that EC is, at least to some degree, sensitive to extinction even though extinction might occur at a slower rate than other forms of Pavlovian conditioning. Hence, EC seems to constitute a particularly persistent form of Pavlovian conditioning with evaluative conditioned effects being even more resistant to extinction in patients with PTSD, as has been shown by Blechert et al. [14] and Wessa and Flor [17]. Such a sustained (differential) negative evaluation of conditioned trauma reminders could maintain the disorder, since situations and stimuli associated with a UCS are disliked and avoided and therefore fear extinction is delayed [14], [49]. Moreover, conditioned negative valence could facilitate the elicitation of negative mood, which, in turn, could make aversive memories more likely [54]. Further supporting a role of persistent negative evaluations for anxiety disorders, Dirikx et al. [55] also reported a relationship between the amount of non-extinguished differential negative evaluations and return of aversively conditioned responses.

We additionally found a marginally significant positive correlation between SCR conditionability and aversive memories during ambulatory assessment. Participants whose previously acquired differential physiological responses remained particularly high during extinction were more likely to experience aversive memories between day 0 and day 2 after the laboratory session. This result is consistent with the similar pattern we found for valence but still needs to be interpreted with caution and replicated due to the asymmetrical and dispersed nature of the bivariate distribution, which could not be normalized (see *Figure 3*, *panel C*). In general, skin conductance response to conditioned stimuli has been shown to be a non-invasive marker for amygdala reactivity in the context of fear learning [56], [57], and persistent differential SCR in the absence of an aversive stimulus can be seen as a failure to extinguish conditioned fear. Paralleling our results, a predictive role of physiological response to trauma-related cues for later posttraumatic symptoms is also supported by a longitudinal study by Suendermann et al. [58]. The authors reported that trauma survivors with PTSD showed greater heart rate responses to standardized trauma reminders 1 month after the trauma compared to those without PTSD and that these responses predicted PTSD symptoms’ severity at 6 month post trauma.

In sum, our results are in line with the cognitive model of PTSD proposed by Ehlers and Clark [3], who suggested the combination of 3 memory processes – strong associative learning (as studied here), poor memory elaboration, and strong perceptual priming – working in conjunction to produce intrusive memories and enable the ease with which it is triggered by trauma reminders. Furthermore, our results open the possibility to extend studies of fear acquisition and extinction in PTSD [14]–[17] to studies of aversive memories and intrusions. Healthy participants in the present study who displayed greater fear conditionability – as indexed by acquired and non-extinguished fear responding – went on to remember more often, longer, and in a more distressing way. Similar mechanisms might be at work in PTSD – this is also supported by a recent study by Lommen et al. [18] who found a predictive role of pre-deployment fear extinction deficits for later posttraumatic symptoms in soldiers. Based on our results, it would be an important further step to implement the conditioned-intrusion paradigm in such a longitudinal high-risk design (while at the same time taking precautions warranted from an ethical point of view). Furthermore, it could be worthwhile to extend this line of research by studying the conditioned-intrusion paradigm using functional neuroimaging and thereby characterizing the underlying neuronal processes (see [59] for a meta-analysis of functional neuroimaging studies of symptom provocation in PTSD patients).

Importantly, the conditioning approach to aversive memories also has therapeutic implications: A specific theory-guided treatment that was suggested by Ehlers and Clark [3] to address the easy triggering of intrusive memories is stimulus discrimination training, in which patients learn to identify subtle sensory triggers as well as to discriminate between the harmless trigger and its present context (“now”) and the stimulus configuration encountered during the trauma (“then”) [3], [5], [6]. Exposure therapy is suggested to further support this discrimination (see e.g., [3]). Furthermore, it enables extinction of conditioned behavioral and psychophysiological reactions to the trauma reminders by establishing a new stimulus-outcome association where the trauma reminder (CS) no longer signals danger [11], [60], [61]. In our study, aversive memories were correlated with conditionability in terms of relatively persistent differential evaluations of the CSs. Such conditioned negative evaluations can be changed by counterconditioning [53], [62], which means pairing the CS with a positively evaluated UCS. However, there is also at least one report of a positive effect of exposure therapy on changing negative evaluations of a feared object [63], but see also [62]. Moreover, an analog study by Ehlers et al. [54] demonstrated the effectiveness of imaginal exposure as well as autobiographical memory elaboration in diminishing differential evaluative conditioning effects and subsequent aversive memories. In sum, further research on possible mechanisms to change conditioned negative evaluations could be a promising future direction to optimize interventions targeting intrusive memories in PTSD.

### Limitations and Directions for Future Research

Some limitations of the present study have to be considered. First, the study used an analog design. Consequently, it remains unclear to what extent the results can be generalized to traumatic events that meet DSM-IV criteria [1]. Furthermore, it is obvious that the aversive memories reported by our participants do not possess the intensity of intrusive memories in PTSD. Even though we included aversive film clips as UCS that were rated highly unpleasant by participants, our stimuli still represent relatively mild stressors in comparison to traumatic events. Note that ethical considerations limit the induction of trauma in laboratory and set inevitable boundaries to the ecological validity of analog studies. Additionally, laboratory fear conditioning experiments require the UCS to be paired with a neutral stimulus (CS^+^) a sufficient number of times to allow the neutral stimulus to become a reliable predictor of the UCS (see e.g., [11], [25]). This may differentiate this laboratory analog from real life traumatic experiences where one exposure is often sufficient to generate a robust association between the aversive event and accompanying neutral stimuli. (On the other hand, however, real-life trauma may involve numerous exposures to threats over hours, days or even years). Second, we did not systematically assess whether participants had previously seen the movies from which the film clips were extracted. Yet, participants’ mean habitual consumption of severely violent film material was rather low and film clips were rated as fairly aversive in the whole sample. Thus, it does not seem to be the case that familiarity with the respective movies interfered with the potential of the film clips to act as a trauma analog. Third, our ambulatory assessment encompassed only day 0 until day 2 after the laboratory session and was thus rather short in comparison to trauma film studies [21]. Further studies might assess participants’ aversive memories over a longer time period. However, mean number, duration, and distress of aversive memories reported on day 2 were fairly small, which was also confirmed by verbal reports participants gave during debriefing. Thus, we believe that a longer standardized assessment of participants’ aversive memories would not have been of essential informational value for our analyses or indicated in terms of ethical considerations. Fourth, it has to be considered that the present sample solely contained female participants, for reasons given above. Further studies should investigate whether our results can be replicated in a male sample and characterize possible gender differences [64], [65]. Fifth, a systematic examination of effects of contingency awareness (CA) on the triggering of aversive memories was beyond the scope of the current study but could still be a promising future direction. Ehlers and Clark [3] concluded that awareness of triggers is not necessary for aversive memories to be elicited. However, it is largely unknown how awareness of triggers exactly influences memory elicitation and how it relates to initial awareness of the presence of a (later trigger) stimulus at traumatic exposure (which is what might correspond best to CA in a fear conditioning context [66]). Preliminary analyses of our data including CA as a between-group factor (see *Figure S2 Supporting information about effects of contingency awareness*) indicate that CA could potentially be a factor worth further investigation. Our analyses on this topic have to be interpreted with caution, though, due to the small number of non-aware participants. Finally, we selected 2 everyday sounds matched for valence and arousal that could both plausibly be paired with violent film clips. However, their valence was not fully matched anymore in the conditioning context (see *Figure S1 Supporting information about effects of assignment of sounds to CS^+^/CS^−^*). Future studies may consider systematically testing the potential of such CSs to be associated with a UCS prior to their implementation in a fear conditioning paradigm.

In conclusion, the conditioned-intrusion paradigm was successful in eliciting fear learning as well as subsequent aversive memories that can be studied in the laboratory as well as in daily life. Our results underline the notion that intrusive memories constitute – at least in part – a non-extinguished conditioned emotional reaction to trauma reminders. Future studies should further characterize the relationship between fear conditionability and aversive memories to allow optimization of clinical interventions for patients suffering from PTSD.

## Supporting Information

Figure S1 **Supporting information about effects of assignment of sounds to CS^+^/CS^−^.**
(DOCX)

Figure S2 **Supporting information about effects of contingency awareness.**
(DOCX)
