# Supplementary material for: Relationship between Fear Conditionability and Aversive Memories: Evidence from a Novel Conditioned-Intrusion Paradigm
Source: PLoS One. 2013 Nov 14;8(11):e79025. doi: 10.1371/journal.pone.0079025 (PMC3828300; doi:10.1371/journal.pone.0079025)
Supplement: Figure S1 — Supporting information about effects of assignment of sounds to CS+/CS−. (DOCX) [file pone.0079025.s001.docx]

***Supporting Information 1***

***“Relationship between fear conditionability and aversive memories: evidence from a novel conditioned-intrusion paradigm”***

Melanie Wegerer ^1^*, Jens Blechert ^1^, Hubert Kerschbaum ^2^, Frank H. Wilhelm ^1^

^1^ Division of Clinical Psychology, Psychotherapy, and Health Psychology

Department of Psychology

University of Salzburg

Salzburg

Austria

^2^ Department of Cell Biology

University of Salzburg

Salzburg

Austria

*Corresponding author:

Melanie Wegerer

Division of Clinical Psychology, Psychotherapy, and Health Psychology

Department of Psychology

University of Salzburg

Salzburg

Austria

E-mail address: melanie.wegerer@sbg.ac.at

**S1 Supporting information about effects of assignment of sounds to CS^+^/CS^-^**

To examine whether one of the two sounds (typewriter or clock) was more easily associable with violent scenes, we added assignment of sounds (typewriter or clock) to CS^+^ and CS^-^ as between-subjects factor to our analysis.

First, we checked if sound assignment had an effect on SCR or behavioral ratings during habituation, acquisition or extinction: In brief (following up on interactions and main effects of higher order MANOVAs and ANOVAs, as described for the main results), for SCR, we found that individuals who had the typewriter as CS^+^ displayed a stronger SCR to the CS^+^ during the first half of extinction, *t*(57)=2.43, *p*=.020, *d*=0.64, than individuals who had the clock as CS^+^. For valence ratings, individuals with the typewriter as CS^+^ (and clock as CS^-^) rated the CS^-^ more negatively during acquisition, *t*(57)=2.68, *p*=.010, *d*=0.70 (no difference for the CS^+^). Finally, for UCS expectancy ratings, we found that individuals who had typewriter as CS^+^ displayed higher UCS expectancy ratings for the CS^-^ during habituation, *t*(57)=5.35, *p*<.001, *d*=1.39. Together, these results do not reveal a consistent pattern across measures in the favor of one of the sound assignments with regard to associability with the aversive films. Furthermore, the overall fear conditioning pattern (with acquisition and later extinction) appeared to be quite similar in individuals who had either typewriter or clock as CS^+^. Similarly, there was neither a main or interaction effect involving sound assignment on aversive memories during the memory triggering task, all *F*s<0.37, all *p*s>.618, nor did sound assignment groups differ in ambulatorily assessed memories, *t*(57)=-1.29, *p*=.202. Regarding correlational analyses, the association of valence conditionability with aversive memories in the CS^+^ condition of the memory triggering task was significant in the group with clock as CS^+^, *ρ*=.50, *p*=.005, while falling below significance in the other group, *ρ*=.24, *p*=.204. The same applies for correlations between valence conditionability and ambulatorily assessed aversive memories: the correlation was significant in the group with clock as CS^+^, *ρ*=.42, *p*=.022 (however, not significant when applying same Bonferroni corrected threshold as in main analyses), but not in the other group, *ρ*=.22, *p*=.256. Correlations between SCR conditionability and ambulatorily assessed aversive memories fell below significance in both groups (all *ρ*s<.33, all *p*s>.073), possibly due to reduced power.
