# Supplementary material for: Relationship between Fear Conditionability and Aversive Memories: Evidence from a Novel Conditioned-Intrusion Paradigm
Source: PLoS One. 2013 Nov 14;8(11):e79025. doi: 10.1371/journal.pone.0079025 (PMC3828300; doi:10.1371/journal.pone.0079025)
Supplement: Figure S2 — Supporting information about effects of contingency awareness. (DOCX) [file pone.0079025.s002.docx]

***Supporting Information 2:***

***“Relationship between fear conditionability and aversive memories: evidence from a novel conditioned-intrusion paradigm”***

Melanie Wegerer ^1^*, Jens Blechert ^1^, Hubert Kerschbaum ^2^, Frank H. Wilhelm ^1^

^1^ Division of Clinical Psychology, Psychotherapy, and Health Psychology

Department of Psychology

University of Salzburg

Salzburg

Austria

^2^ Department of Cell Biology

University of Salzburg

Salzburg

Austria

*Corresponding author:

Melanie Wegerer

Division of Clinical Psychology, Psychotherapy, and Health Psychology

Department of Psychology

University of Salzburg

Salzburg

Austria

E-mail address: melanie.wegerer@sbg.ac.at

**S2 Supporting information about effects of contingency awareness**

The relatively small subsample of 11 contingency unaware participants (18.6%) mandates a cautious interpretation of the following between-group analyses. An *à priori* investigation of effects of contingency awareness (CA) would require specific adaptations of CS presentation (i.e., increasing the proportion of non-aware participants, e.g., by more complex contingencies involving several CSs, inclusion of distractor stimuli, or application of difficult-to-distinguish CS pairs such as highly similar visual cues [1-3]) and a better CA assessment (see e.g., [4] for an extensive review of difficulties of assessing CA). Up to now, the role of CA for differential conditioning is still discussed controversially (see e.g., [1,2,5]). Furthermore, from the perspective of PTSD symptomatology which motivated the present research, it is rather unclear how CA influences (later) triggering of aversive memories. Importantly, Ehlers and Clark [6] concluded that awareness of triggers is not necessary for aversive memory elicitation. However, it is largely unknown how being aware or unaware of triggers exactly influences triggering of aversive memories. Furthermore, it remains unclear how unawareness of triggers *in the moment of memory elicitation* relates to initial awareness of the presence of the respective stimuli *in the moment of traumatic exposure* (which is what probably corresponds best to what is known as CA in a fear conditioning context [4]). Based on these methodological and theoretical considerations and the fact that we aimed at investigating the general relationship between fear conditionability and aversive memories – irrespective of CA – both participants with and without CA were maintained in the main analyses (see main text). However, to encourage further research on this important topic we reanalyzed our data, including CA as a between-participants factor. For sake of brevity, we focus on measures for which an overall CS-type x Time MANOVA yielded significant main effects or interaction effects involving CA.

**Fear conditioning**

*Table S1* displays mean values and standard deviations for behavioral ratings and SCRs during the fear conditioning task for contingency aware and non-aware participants.

*Habituation*: During habituation, there was neither a main effect of CA nor a significant interaction involving CA on behavioral ratings or SCR, all *F*s<1.90, *p*>.140.

*Acquisition*: Following up on a significant MANOVA main and interaction effect of CA (all *F*s(3,55)>4.82, *p*<.005, *η^2^*>.21), separate measure wise ANOVAs for ratings of fear, valence and UCS-expectancy similarly yielded CA main effects, all *F*s(1,57)>5.17, *p*<.027, *η^2^* >.08, which were modulated by a CS-type x CA interaction, all *F*s(1,57)>12.14, *p*<.001, *η^2^* >.18. *Post-hoc* *t*-tests revealed that participants with CA displayed significantly higher (i.e., more aversive) ratings for the CS^+^ as compared to the CS^-^, all *t*s(47)>9.68, *p*<.001, *d*>1.83, whereas differences between CS^+^ and CS^-^ were not significant in participants without CA, all *t*s(10)<2.11, *p*>.061. This pattern appeared to be caused by higher ratings for the CS^-^ in non-aware participants as compared to aware participants, all *t*s(57)>4.09, *p*<.001, *d*>1.30. For SCR, there was a CS-type x CA interaction, *F*(1,57)=4.29, *p*=.043, *η^2^=.*07, revealing that aware participants displayed significantly higher SCRs to the CS^+^ as compared to the CS^-^, *t*(47)=3.72, *p*=.001, *d*=0.27, whereas there was no such difference in participants without CA, *t*(10)=-0.65, *p*=.531.

*Extinction*: During extinction, the MANOVA for behavioral ratings revealed a significant CS-type x CA as well as a CS-type x CA x Time interaction (all *F*s(3,55)>3.68, *p*<.017, *η^2^*>.17) which were confirmed for each single rating scale by follow-up ANOVAs (CS-type x CA interactions: all *F*s(1,57)>7.75, *p*<.007, *η^2^*>.12; CS-type x CA x Time interactions: all *F*s(1,57)>4.24, *p*<.044, *η^2^*>.07). All ratings were significantly higher (more aversive) for the CS^+^ as compared to the CS^-^ in the aware, all *t*s(47)>5.99, *p*<.001, *d*>1.12, but not in the non-aware group, all *t*s(10)<2.11, *p*>.061. Moreover, valence and fear ratings for CS^+^ significantly dropped between first and second half of extinction in the aware group, all *t*s(47)>4.95, *p*<.001, *d*>0.44, whereas that was not the case in the non-aware group, all *t*s(10)<1.40, *p*>.193. Ratings of UCS expectancy, however, significantly dropped for both CS^+^ and CS^-^ in the non-aware group, all *t*s(10)>2.71, *p*<.022, *d*>0.36, but only for CS^+^ in the aware group, *t*(47)=5.60, *p*<.001, *d*=0.76. For SCR, there was neither a CA main effect nor any interaction involving CA, all *F*s(1,57)<1.79, *p*>.186.

**Memory triggering task**

*Table S2* displays mean values and standard deviations for aversive memories, state anxiety, and SCL for the memory triggering task for contingency aware and non-aware participants.

*IMQ sum score*: There was a significant interaction effect of CS-type x CA, *F*(2,114)=4.18, *p*=.025, *η^2^=.07,* but no main effect of CA, *F*(1,57)=0.49, *p*=.486, for aversive memories during the memory triggering task. Participants without CA descriptively displayed the same response pattern as participants with CA with strongest (i.e., more, longer and more distressing) aversive memories during the CS^+^ cue condition and still stronger memories during the CS^-^ cue condition as compared to the no-cue condition. However, whereas differences in aversive memories between conditions were significant in the contingency aware group, all *t*s(47)>3.92, *p*<.001, *d*>0.43 , this was not the case in the non-aware group, all *t*s(10)<1.21, *p*>.256, which may in part be due to the smaller sample size.

*STAI state anxiety*: For STAI state anxiety, again, there was a significant interaction of CS-type x CA, *F*(2,112)=3.99, *p*=.021, *η^2^*=.07, but no main effect of CA, *F*(1,56)=0.02, *p*=.883. Whereas aware participants’ state anxiety significantly differed between different conditions, all *t*s(46)>2.91, *p*<.006, *d*>0.23, that was not the case for non-aware participants, all *t*s(10)<1.65, *p*>.129.

*SCL*: For SCL, there was neither a main effect for CA nor a CS-type x CA interaction, all *F*s<1.97, *p*>.145.

**Ambulatory assessment of aversive memories**

Participants with and without CA did not significantly differ in the extent (number, duration, and distress as assessed by the Intrusion Memory Questionnaire) of aversive memories that they reported on day 0-2 after the laboratory session (aware participants: *MW*=49.76, *SD*=20.81; non-aware participants: *MW*=51.05, *SD*=15.28; *t*(57)=0.19, *p*=.847).

**Supporting Table Captions**

*Table S1*: Means and standard deviations for behavioral ratings and SCRs during the fear conditioning task for contingency aware (CA) and non-aware (non-CA) participants.

*Table S2*: Means and standard deviations for aversive memories, state anxiety, and SCL during the memory triggering task for contingency aware (CA) and non-aware (non-CA) participants.

**Table S1**

|  |  | **Hab(1)** | | **Hab(2)** | |  | **Acq(1)** | | **Acq(2)** | |  | **Ext(1)** | | **Ext(2)** | |
| --- | --- | --- | --- | --- | --- | --- | --- | --- | --- | --- | --- | --- | --- | --- | --- |
|  |  | **CA** | **non-CA** | **CA** | **non-CA** |  | **CA** | **non-CA** | **CA** | **non-CA** |  | **CA** | **non-CA** | **CA** | **non-CA** |
|  |  | *M* (*SD*) | *M* (*SD*) | *M* (*SD*) | *M* (*SD*) |  | *M* (*SD*) | *M* (*SD*) | *M* (*SD*) | *M* (*SD*) |  | *M* (*SD*) | *M* (*SD*) | *M* (*SD*) | *M* (*SD*) |
| Valence | CS^+^ |  |  | 48.06 (19.74) | 59.98  (26.65) |  | 79.16  (19.99) | 79.08  (21.22) | 83.66  (17.36) | 83.83  (20.77) |  | 74.43  (21.12) | 71.70  (32.79) | 65.29  (20.10) | 73.51  (24.61) |
|  | CS^-^ |  |  | 53.42  (19.88) | 55.77  (16.56) |  | 39.65  (26.28) | 70.43  (31.08) | 38.16  (27.66) | 75.61  (26.85) |  | 42.89  (28.66) | 77.59  (23.48) | 44.59  (25.71) | 69.66  (26.01) |
| Fear | CS^+^ |  |  | 18.82  (22.67) | 30.46  (29.42) |  | 74.66  (24.80) | 66.96  (35.93) | 78.59  (23.22) | 76.89  (33.61) |  | 70.97  (24.16) | 68.01  (35.28) | 59.59  (25.58) | 64.72  (35.43) |
|  | CS^-^ |  |  | 19.94  (23.52) | 32.35  (30.48) |  | 30.92  (27.76) | 66.39  (29.09) | 33.28  (26.24) | 71.68  (33.20) |  | 34.22  (30.64) | 66.90  (33.75) | 32.39 (29.06) | 59.76  (35.40) |
| UCS expectancy | CS^+^ |  |  | 44.18  (25.53) | 65.38  (26.32) |  | 87.49  (16.62) | 82.06  (22.84) | 93.27  (11.47) | 86.22  (22.96) |  | 78.58  (21.92) | 74.97  (28.14) | 58.80  (29.84) | 64.06  (27.44) |
|  | CS^-^ |  |  | 49.24  (28.04) | 55.08  (24.33) |  | 32.18  (29.36) | 68.14  (32.87) | 26.90  (30.72) | 79.77  (26.33) |  | 30.35  (31.40) | 71.08  (26.82) | 29.97  (31.86) | 61.22  (28.64) |
| SCR | CS^+^ | 0.0506  (0.0801) | 0.0346  (0.0659) | 0.0285  (0.0637) | 0.0031  (0.0192) |  | 0.1858  (0.2362) | 0.0523  (0.0742) | 0.1878  (0.1771) | 0.1147  (0.0943) |  | 0.1664  (0.2169) | 0.0724  (0.0808) | 0.0835  (0.1262) | 0.0484  (0.0528) |
|  | CS^-^ | 0.0703  (0.1103) | 0.0477  (0.0385) | 0.0362  (0.0743) | 0.0112  (0.0274) |  | 0.1466  (0.2247) | 0.1096  (0.1379) | 0.1244  (0.1580) | 0.0794  (0.0864) |  | 0.1039  (0.1464) | 0.0540  (0.0726) | 0.0605  (0.0913) | 0.0209  (0.0383) |

*Note: Hab:* Habituation*; Acq:* Acquisition; *Ext:* Extinction; *SCR*: skin conductance response given as ln(1+SCR) in μS*.* Note that there are no values for ratings of valence, fear, and UCS expectancy for the first half of habituation since behavioral ratings were only collected at the end of the habituation period.

**Table S2**

|  | **CS^+^ cue cond.** | |  | **CS^-^ cue cond.** | |  | **No-cue cond.** | |
| --- | --- | --- | --- | --- | --- | --- | --- | --- |
|  | **CA** | **non-CA** |  | **CA** | **non-CA** |  | **CA** | **non-CA** |
|  | *M* (*SD*) | *M* (*SD*) |  | *M* (*SD*) | *M* (*SD*) |  | *M* (*SD*) | *M* (*SD*) |
| IMQ – Sum score | 65.11 (30.00) | 48.64 (16.86) |  | 49.51 (28.69) | 45.21 (14.91) |  | 38.30 (23.07) | 43.40 (15.17) |
| State anxiety | 46.50 (13.11) | 45.91 (9.38) |  | 43.60 (12.40) | 41.82 (8.60) |  | 40.06 (11.23) | 44.73 (9.18) |
| SCL | 1.8495 (0.2885) | 1.9280 (0.3752) |  | 1.8463 (0.2936) | 1.8946 (0.3938) |  | 1.8368 (0.2898) | 1.8977 (0.3760) |

*Note:* *cond.*: condition; *IMQ sum score*: values represent sum scores of the IMQ (Intrusion Memory Questionnaire) in T-scores;

*State anxiety*: assessed by STAI state scale; *SCL*: skin conductance level given as ln(1+SCL) in μS*.*

**References**

1. Schultz DH, Helmstetter FJ (2010) Classical conditioning of autonomic fear responses is independent of contingency awareness. J Exp Psychol Anim B 36: 495-500.

2. Tabbert K, Merz CJ, Klucken T, Schweckendiek J, Vaitl D, et al. (2011) Influence of contingency awareness on neural, electrodermal and evaluative responses during fear conditioning. Soc Cogn Affect Neurosci 6: 495-506.

3. Singh K, Dawson ME, Schell AM, Courtney CG, Payne AFH (2013) Can human autonomic classical conditioning occur without contingency awareness? The critical importance of trial sequence. Biol Psychol 93: 197-205.

4. Lovibond PF, Shanks DR (2002) The role of awareness in Pavlovian conditioning: empirical evidence and theoretical implications. J Exp Psychol Anim B 28: 3-26.

5. Pearson J (2012) Associative learning: Pavlovian conditioning without awareness. Curr Biol 22: R495-R496.

6. Ehlers A, Clark DM (2000) A cognitive model of posttraumatic stress disorder. Behav Res Ther 38: 319-345.
